# Predictors of nonresponse to treatment and low adherence to internet-based cognitive behavioral therapy in depressed/anxious women facing the couple’s fertility problems: a secondary analysis of a randomized control trial

**DOI:** 10.1186/s12888-023-05484-3

**Published:** 2024-01-10

**Authors:** Shiva Shafierizi, Zahra Basirat, Fatemeh Nasiri-Amiri, Farzan Kheirkhah, Zahra Geraili, Hajar Pasha, Mahbobeh Faramarzi

**Affiliations:** 1 Student Research Committee, Babol University of Medical Sciences, Babol, Iran; 2 Department of Obstetrics and Gynecology, Infertility and Reproductive Health Research Center, Health Research Institute, Babol University of Medical Sciences, Babol, Iran; 3 Department of Midwifery, Infertility and Reproductive Health Research Center, Health Research Institute, Babol University of Medical Sciences, Babol, Iran; 4 Department of Psychiatry, Social Determinants of Health Research Center, Health Research Institute, Babol University of Medical Sciences, Babol, Iran; 5 Social Determinants of Health Research Center, Health Research Institute, Babol University of Medical Sciences, Babol, Iran; 6 Department of General Courses, Infertility and Reproductive Health Research Center, Health Research Institute, Babol University of Medical Sciences, Babol, Iran

**Keywords:** Nonresponse, Low adherence, Internet-based cognitive behavioral therapy, Depression, Anxiety, Infertility

## Abstract

**Background:**

The study aimed to examine the predictors of treatment nonresponse and low adherence to Internet-based cognitive behavioral therapy and face-to-face therapy for treating depression and anxiety in women facing the couple’s fertility problems.

**Methods:**

This is a secondary analysis based on a previous randomized controlled trial including 152 depressed/anxious women facing the couple’s fertility problems. The study defines low adherence as receiving less than 4 sessions (out of 8 sessions). Nonresponse to treatment refers to a < 50% reduction in the anxiety and depression total scores.

**Results:**

A high level of anxiety/depression score before psychotherapy increases the risk of nonresponse to both Internet-based and face-to-face psychotherapies by 1.4 to 2 times in women facing the couple’s fertility problems after the treatment and in the 6-month follow-up. However, 4 factors, including diagnosis of mixed anxiety and depression, low education level, long marriage duration, and infertility caused by mixed female/male factors, reduced the risk of nonresponse to psychotherapies.

**Conclusion:**

Women facing the couple’s fertility problems with high depression and anxiety scores are at risk of poor prognosis in response to psychotherapy. Psychologists and healthcare providers of infertility centers should pay more attention to the timely identification and referral of depressed/anxious patients to psychologists.

**Supplementary Information:**

The online version contains supplementary material available at 10.1186/s12888-023-05484-3.

## Introduction

Infertility is characterized as the inability to conceive after 1 year or more of regular unprotected intercourse [1]. Furthermore, infertility itself can cause psychological [2], social, and behavioral consequences [3, 4]. Infertile individuals often experience negative emotions, such as anxiety, depression, and frustration, which can affect overall well-being, treatment success, and willingness to continue with treatment [3, 5, 6]. Moreover, psychological problems before or during assisted reproductive therapy (ART) can potentially reduce the chances of pregnancy [7]. The importance of psychosocial interventions for infertile individuals has been recommended by several studies [7]. There is a general agreement in the literature on the efficacy of Internet-based and face-to-face cognitive behavioral therapy (CBT) for infertile patients [8–11].

Over the last 2 decades, Internet-based psychotherapies have been developed and become an important part of routine healthcare settings. One of the most widely used forms is Internet-based cognitive behavioral therapy (ICBT) [12]. The efficacy of ICBT has been assessed in approximately 300 randomized controlled trials and several systematic reviews and meta-analyses, indicating its effectiveness for many mental and physical problems [13]. However, most studies have focused on the efficacy of ICBT while paying less attention to the lack of effect.

Nonresponse is defined as the failure to achieve clinically significant symptom reductions after treatment [14]. There is currently no consensus on how to categorize patients as nonresponders. Looking more closely at how this was defined in specific clinical trials indicated that most studies applied the reliable change index, a clinical cut-off or change from baseline [14, 15]. Unfortunately, few studies have provided empirical insight into the nonresponse occurrence and predictors. Boettcher et al. (2014) conducted a randomized control trial on 133 patients diagnosed with social anxiety disorder. They found that nonresponse to ICBT was frequent, with 32–50% for social anxiety measures and 57–90% for secondary outcomes at post-assessment [16]. A recent meta-analysis of nonresponse in ICBT reported that of the 2,118 patients, 26.8% were classified as nonresponders [15]. A better understanding of the nonresponse rate and its predictors may guide the choice or development of individualized treatment approaches, aiming to increase response rates among those affected [17].

Since many patients are considered nonresponders, it is important to find the risk factors and determinants of this category. Evidence on nonresponse predictors has been inconsistent [15, 17]. Several studies have identified various patient variables, such as sociodemographic, comorbidity, baseline symptom severity, adherence, and personality, as predictors of treatment outcome [18–20]. For example, previous studies suggested that high pre-treatment symptom severity [14, 17] and comorbidity [14] are associated with poorer response, while other studies showed that having more symptoms, higher education, and being female were predictors of treatment improvement [21, 22]. However, most studies focus on the responders rather than explicitly examining the nonresponders. This is an essential step for researchers and clinicians to identify nonresponders to understand how treatment could be provided to avoid treatment failures [23].

Internet-based treatments present issues with a high degree of nonadherence [24]. Dropout during treatment varied from 0 to 78% in online interventions for psychological disorders. The literature shows that therapist-guided programs contribute to higher adherence, with average levels of adherence estimated at 72% [25]. A previous study evaluating the effectiveness and acceptance of Internet-based treatment for infertile patients reported that out of the 52 participants in the intervention group, 50% completed the entire self-help guide (consisting of 13 sessions), while 28.8% completed 50% or more of the guide [26]. A growing body of research has introduced various predictors of ICBT adherence, such as sex, age, educational level, and baseline symptom severity, to be related to adherence treatment [27–30]. Karyotaki et al. (2015), in a meta-analysis, demonstrated that male sex, lower educational level, and comorbid anxiety symptoms significantly decreased the odds of adherence, while older age increased the risk of adherence [27]. Besides, knowing adherence determinants is essential as higher adherence to Internet interventions has been found to be associated with better outcomes [31]. A study reported that higher levels of adherence were associated with a greater reduction in symptoms of depression and anxiety [32].

Few studies have identified the predictors of nonresponse and nonadherence to ICBT for anxiety and depression [15, 16]. As ICBT is becoming more common in routine mental health services, it could be beneficial to determine why some patients do not respond to online intervention. This category of patients has often been neglected in research as most randomized controlled studies focus on the responders and provide insufficient information on harmful treatment effects for some patients. To the best of our knowledge, this is the first study to examine predictors of nonresponse to treatment and low adherence of women facing the couple’s fertility problems for ICBT. The current study aimed to investigate treatment nonresponse, low adherence occurrence, and the predictors.

## Materials and methods

### Study design

The current study is a secondary analysis of a multicenter, single-blinded, non-inferior, 2-parallel randomized controlled trial (RCT), which examined the efficacy of an ICBT program (Peaceful Mind) for adjustment disorders among women facing the couple’s fertility problems. This study was conducted from September 2020 to July 2021. In the previous study, participants were randomized to a therapist-guided ICBT group or face-to-face CBT group. The protocol of the original study was registered in the Iranian Registry of Clinical Trials (IRCT20110228005931N8). The research design and main results have been described elsewhere [8]. We briefly summarize the method here.

### Participants

The participants were women facing the couple’s fertility problems who received ART methods from Fatemeh Zahra Infertility and Reproductive Health Research Center and Mehregan Private Hospital (Iran). The inclusion criteria for the trial were: 18 years of age or older, education level higher than elementary school, Internet access, ability to use computers, mobile phones, etc., currently undergoing outpatient psychotherapy, not taking psychiatric medications in the past 3 months, providing written informed consent, and being diagnosed with an adjustment disorder according to the Diagnostic and Statistical Manual of Mental Disorders, 5th Edition [33]. We excluded patients with severe psychiatric disorders (based on the clinical interview for DSM-5 (SCID-5-CV)) and developmental disorders (based on the patients’ reports).

### Procedure

Obstetricians, gynecologists, and midwives of 2 infertility centers referred women seeking ARTs to the research team to evaluate the inclusion/exclusion criteria. Eligible women were referred for a clinical interview with a psychologist or a psychiatric resident. Telephone interviews were conducted for patients unwilling to visit the clinic (because the research was conducted during the COVID-19 pandemic). Out of 261 infertile women interviewed, 109 patients were excluded from the study. Random allocation by the permuted block technique was used to allocate the remaining participants into ICBT (n = 76) or CBT (n = 76) interventions at a 1:1 ratio. In the current study, 152 participants were selected; those who were allocated to intervention groups (ICBT or F2F CBT) (patients having recruited and completed treatment between September 2020 and January 2021).

All the participants filled their assessments through a website (DigiSurvey), including the Adjustment Disorder New Module-20 (ADNM-20), Hospital Anxiety and Depression Scale (HADS), Fertility Adjustment Scale, Fertility Problem Inventory, Cognitive Therapy Awareness Scale, and Automatic Thought Questionnaire before the intervention, after the treatment (eighth week), in the 3-month follow-up, and in the 6-month follow-up after the treatment (Readers can refer to Shafierizi et al. [8] for a complete description of the recruitment process, randomization, and data collection procedure).

### Intervention groups

The treatment was conducted in a face-to-face and online therapist-guided program in eight 50-minute individual sessions by 2 therapists. The session content, psychoeducation, and face-to-face exercises were the same as the online sessions. The modules included psychoeducation, principles of CBT, restructuring techniques, behavioral techniques, changing schemas, and reviewing goals. Face-to-face CBT was performed by a professional psychologist (MF) in individual sessions. In addition, an assistant (SS) participated in sessions and helped with the patients’ psychoeducation, supported the patients in completing their assignments, and provided feedback. In therapist-guided ICBT, participants received 20-minute guided therapy via phone weekly. The online treatment was administered on the Peaceful Mind website. In previous studies, this program has been demonstrated to be effective for psychological disorders among women facing the couple’s fertility problems [8, 9]. The therapist could manage patients’ registration and monitor their activities in the therapist panel. The participants could access the patient panel by entering their usernames and passwords (www.peacefulmindme.com). The website included a library, videos from a psychologist, videos from fictitious characters, photos, texts, the guide’s voice, quizzes, and mental exercises that help the patients through self-help methods and understanding the content. The content of the session is presented in Table S1 (See Supplementary Table S1**).**

Patients received 20-minute phone-guided therapy sessions once a week, in addition to Internet therapy. The sessions were conducted by a psychologist with ICBT experience (MF) and an assistant psychologist (SS). The therapists reviewed concepts and assignments and answered the questions. The assistant reminded the participants to attend sessions, supported exercises and provided motivation. No face-to-face contact occurred. Text reminders were sent if the patients did not log in for 1 week. Except for some patients who complained about the slow Internet speed, there were no other technical problems on the website. More details about the interventions are published in our previous studies [8, 9].

### Low adherence and nonresponse definition

Adherence was defined as receiving at least 4 sessions. Therefore, a participant was considered to belong to the high-adherence group if they completed 4 or more sessions, and a patient who completed less than 4 sessions was considered a low-adherence participant. The number of sessions seen by the patient in the ICBT group was recorded in the therapist’s panel. Therefore, through the website, the therapist could monitor how many sessions the patient completed at the end of the treatment. In addition, nonresponse to treatment means a < 50% reduction in the HADS total score.

### Measurements

#### Adjustment disorder new module-20 (ADNM-20)

The ADNM-20 is a self-report tool that evaluates the severity of adjustment disorder symptoms on a 4-point scale, from 1 (never) to 4 (often) [34]. This scale consists of 2 core subscales, Preoccupation and Failure to Adapt, and 4 accessory sub-scales, namely Avoidance, Depressive Mood, Anxiety, and Impulsive Disturbance. Symptom severity can be evaluated by the sum score of all items (total score ranged from 20 to 80). A total score of ADNM-20 > 47.5 indicated the high severity of adjustment disorder symptoms [35]. This study used the Persian version of the ADNM-20 [36]. The Cronbach’s alpha of the questionnaire was 1.57.

#### Hospital anxiety and depression scale (HADS)

The 14-item self-report HADS contains 7 items evaluating anxiety and 7 measuring depression [37]. Each question was scored on a 4-point Likert scale ranging from 0 to 3 (the range of the total score = 0–42). For both subscales, the cut-off point > 7 indicated the symptom of depression or anxiety. A total score of HADS > 14 demonstrated depressive and anxiety symptoms. The validated Persian version was used in the study [38]. Cronbach’s alpha for HADS anxiety and depression sub-scales was 0.78 and 0.86, respectively.

#### Fertility adjustment scale

Fertility Adjustment Scale was developed by Glover et al. (1999) [39]. This scale evaluates the infertility adjustment through 12 questions. Each item is scored on a 6-point Likert scale from 1 (strongly disagree) to 6 (strongly agree). The total score is obtained by summing the scores of all items (range = 12–72), and a high score means a low adjustment level. The validity and reliability of the Persian version were examined by Tiyuri et al. (2018) [40]. The Cronbach’s alpha value of 0.68 indicates moderate reliability.

#### Fertility problem inventory

This 46-item tool was designed by Newton et al. (1999) to measure infertility stress levels [41]. It has 5 subscales, namely sexual concern, social concern, relationship concern, need for parenthood, and rejection of childfree lifestyles. The response format is a 4-point Likert scale, ranging from 1 (strongly disagree) to 6 (strongly agree). The total score is obtained from the sum of the scores of all items, with a range of 46–276, and higher scores define higher stress levels. We used the validated Persian version [42]. Cronbach’s alpha for all sub-scales was more than 0.7, and the overall integrity was found to be 0.87.

#### Cognitive therapy awareness scale

The Cognitive Therapy Awareness Scale (CTAS) was used to evaluate the participants’ levels of knowledge about CBT principles and methods. This questionnaire contains 40 true/false items, and 10 questions have 4 options. The correctness or incorrectness of each option must be determined, and the range of total scores is from 0 to 40 [43].

#### Automatic thought questionnaire

The Automatic Thoughts Questionnaire (ATQ) was given to the patients to measure the frequency of negative thoughts. For this 30-item questionnaire, each item is scored based on a 5-point scale from 1 (never) to 5 (always) [44]. The total score is obtained from the sum of the items and ranges from 30 to 150. We used the valid Persian version of ATQ [45]. The internal consistency of ATQ-Persian was excellent, with a Cronbach’s alpha of 0.96.

### Statistical analysis

Data analysis was conducted using SPSS v. 22.0 (IBM Corp., Armonk, NY, USA). Descriptive statistics were conducted to describe the psychodemographics of the sample. The *t*-test was used to compare the means of demographic variables and psychological profiles based on adherence to treatment and response to treatment in each intervention group. We also used the chi-square test to compare the differences between categorical variables of intervention groups regarding adherence to treatment and response to treatment. Finally, we used multiple regression logistic models with hospital anxiety and depression total score, education level, cause of infertility, principal diagnosis, marriage duration as independent variables, and nonresponse and low adherence to treatment as dependent variables, in two separate models. *P*-value < 0.05 was considered significant.

We determined the sample size using a previous study [46]. PASS v. 11 software was used for a non-inferiority analysis with a margin of 1.5 for depression score and standard deviation (SD) = 3. Bonferroni’s method was employed (α = 0.05 and 80% power). We calculated a sample of 152 with a 15% attrition rate. The sample size equation is as follows:

$$\frac{{{\sigma ^{\rm{2}}}{{({{\rm{z}}_{{\rm{1 - }}\frac{\alpha }{{\rm{2}}}}}{\rm{ + }}\,{{\rm{z}}_{{\rm{1 - }}\beta }})}^{\rm{2}}}}}{{{{({\mu _{\rm{1}}}{\rm{ - }}{\mu _{\rm{2}}})}^{\rm{2}}}}}$$

## Results

The participants’ mean age was 31.5 ± 5.5 years. Most of the women facing the couple’s fertility problems were homemakers (75%), and just over half of them were urban residents (55.3%) and had a high school diploma or lower education (53.6%). Among the principal diagnoses, the prevalence of adjustment disorder with depressed mood was 37.5%, adjustment disorder with anxious mood was 21.7%, and adjustment disorder with mixed anxiety and depressed mood was 40.8%. Furthermore, the mean score of the adjustment disorder of women facing the couple’s fertility problems was slightly high (M = 54.9 ± 11.9 of the possible range of 20–80). In addition, the mean of total anxiety and depression was M = 16.8 ± 7.9 (the possible range: 0–42), demonstrating that anxiety and depression symptoms were higher than moderate.

Table 1 shows the demographic and clinical characteristics of both groups regarding adherence to treatment. The result of the *t*-test indicated that the mean of fertility adjustment problems (*P* = 0.042) and infertility stress (*P* = 0.027) were significantly higher in patients with low adherence in the CBT group. However, for demographic and psychological variables, including age, education, place of residence, job, infertility duration, cause of infertility, principal diagnosis, marriage duration, adjustment disorder, psychological distress, cognitive therapy awareness, and automatic thought, there was no significant difference between patients with low and high adherence in the two intervention groups.

**Table 1:** Characteristics of the study population based on adherence to treatment in two groups of psychotherapy

variables	Total	CBT			ICBT		
		Low adherence (n = 22)	High adherence (n = 54)	*p*-value	Low adherence (n = 9)	High adherence (n = 67)	*p*-value
Age, mean ± Sd	31.5 ± 5.5	32.4 ± 6.3	33.1 ± 5.5	0.681	32.2 ± 4.6	30.1 ± 5.1	0.229
Education, n (%)							
≤Diploma University	81 (53.6) 70 (46.4)	12 (54.5) 10 (27.8)	28 (51.9) 26 (72.2)	1.000	3 (33.3) 6 (66.7)	38 (57.6) 28 (42.4)	0.285
Place of residence, n (%)							
City Town	84 (55.3) 68 (44.7)	11 (50) 11 (50)	29 (53.7) 25 (46.3)	0.805	6 (66.7) 3 (33.3)	38 (56.7) 29 (43.3)	0.726
Job, n (%)							
Unemployed Employee	114 (75) 38 (25)	17 (77.3) 5 (22.7)	42 (77.8) 12 (22.2)	1.000	7 (77.8) 2 (22.2)	52 (77.6) 15 (22.4)	1.000
Duration of infertility, n (%)							
≤4 ≥5	64 (42.7) 86 (57.3)	10 (45.5) 12 (54.5)	18 (34) 35 (66)	0.434	5 (62.5) 3 (37.5)	31 (46.3) 36 (53.7)	0.469
Caused of infertility, n (%)							
Female factors Male factors Female and male factors Unknown	27 (17.9) 35 (23.2) 49 (32.5) 40 (26.5)	2 (9.1) 7 (31.8) 8 (36.4) 5 (22.7)	8 (14.8) 16 (29.6) 14 (25.9) 16 (29.6)	0.736	3 (33.3) 3 (33.3) 0 3 (33.3)	14 (21.2) 9 (13.6) 27 (40.9) 16 (24.2)	0.095
Principal diagnosis, n (%)							
with depressed mood with anxious mood With mixed anxiety and depressed mood	57 (37.5) 33 (21.7) 62 (40.8)	8 (36.4) 7 (31.8) 7 (31.8)	22 (40.7) 14 (25.9) 18 (33.3)	0.868	4 (44.4) 3 (33.3) 2 (22.2)	23 (34.3) 9 (13.4) 35 (52.2)	0.158
Duration of marriage, mean ± Sd	8.0 ± 4.2	8.1 ± 5.1	8.8 ± 4.6	0.573	6.7 ± 4.3	7.5 ± 3.5	0.568
Infertility duration, mean ± Sd	5.7 ± 3.5	6.5 ± 4.9	6.2 ± 3.4	0.764	4.7 ± 3.5	5.2 ± 2.9	0.639
Adjustment disorder, mean ± Sd	54.9 ± 11.9	58.9 ± 9.8	53.1 ± 12.3	0.057	53.3 ± 9.4	55.3 ± 12.3	0.638
Hospitalized anxiety and depression, mean ± Sd	16.8 ± 7.9	20.3 ± 7.7	16.9 ± 7.5	0.084	13.0 ± 3.8	16.0 ± 8.4	0.085
Fertility adjustment scale, mean ± Sd	46.2 ± 8.2	49.0 ± 8.2	44.8 ± 7.9	0.042	45.5 ± 11.0	46.4 ± 8.0	0.770
Fertility problem inventory, mean ± Sd	152.4 ± 32.7	167.4 ± 30.6	150.8 ± 28.5	0.027	139.8 ± 33.4	150.4 ± 35.6	0.402
Cognitive therapy awareness scale, mean ± Sd	18.1 ± 7.3	18.6 ± 4.7	17.5 ± 7.2	0.522	19.5 ± 9.0	18.1 ± 8.0	0.639
Automatic thought questionnaire, mean ± Sd	69.0 ± 28.8	70.7 ± 26.5	70.0 ± 25.8	0.916	54.3 ± 19.7	69.5 ± 26.3	0.099

The relationship between response to treatment and the demographic and psychological variables of the ICBT group at three points in time is reported in Table 2. Our study showed that nonresponse to CBT and ICBT was 68.4% and 71.1% at the end of the treatment, 67.1% and 61.8% in the 3-month follow-up, and 67.1% and 68.4% in the 6-month follow-up, respectively. Adherence to treatment (receiving at least 4 sessions) was 88.2% in the ICBT group and 71.1% in the CBT group. Attrition rates in the ICBT group were 14.4% and 15.7% in 3- and 6-month follow-ups, respectively. In the CBT group, attrition rates were 17.1% in the 3-month follow-up and 19.7% in the 6-month follow-up.

**Table 2:** Comparison of the demographic and clinical characteristics of the women facing couple’s fertility problem regarding response to treatment in ICBT group

variables	Post-test			3-month follow-up			6-month follow-up		
	Response (n = 22)	non-response (n = 54)	*P*-value	Response (n = 29)	non-response (n = 47)	*P*-value	Response (n = 24)	non-response (n = 52)	*P*-value
Education									
≤Diploma University	14 (63.6) 8 (36.4)	27 (50.9) 26 (49.1)	0.445	17 (58.6) 12 (41.4)	24 (52.2) 22 (47.8)	0.639	15 (62.5) 9 (37.5)	26 (51) 25 (49)	0.457
Job									
Unemployed Employee	19 (86.4) 3 (13.6)	40 (74.1) 14 (25.9)	0.365	24 (82.8) 5 (17.2)	35 (74.5) 12 (25.5)	0.572	20 (83.3) 4 (16.7)	39 (75) 13 (25)	0.558
Place of residence									
City Town	15 (68.2) 7 (31.8)	29 (53.7) 25 (46.3)	0.310	19 (65.5) 10 (34.5)	25 (53.2) 22 (46.8)	0.334	15 (62.5) 9 (37.5)	29 (55.8) 23 (44.2)	0.625
Caused of infertility									
Female factors Male factors Female and male factors Unknown	4 (18.2) 4 (18.2) 10 (45.5) 4 (18.2)	13 (24.5) 8 (15.1) 17 (32.1) 15 (28.3)	0.621	5 (25.5) 5 (25.5) 12 (42.9) 6 (21.4)	12 (25.5) 7 (14.9) 15 (31.9) 13 (27.7)	0.704	6 (26.1) 3 (13) 11 (47.8) 3 (13)	11 (21.2) 9 (17.3) 16 (30.8) 16 (30.8)	0.306
Principal diagnosis									
depressed mood anxious mood mixed anxiety and depressed mood	7 (31.8) 3 (13.6) 12 (54.5)	20 (37) 9 (16.7) 25 (46.3)	0.807	8 (27.6) 6 (20.7) 15 (51.7)	19 (40.4) 6 (12.8) 22 (46.8)	0.442	6 (25) 3 (12.5) 15 (62.5)	21 (40.4) 9 (17.3) 22 (42.3)	0.258
Age	29.5 ± 4.4	30.5 ± 5.3	0.438	28.8 ± 4.4	31.2 ± 5.2	0.045	28.7 ± 3.9	31.0 ± 5.3	0.038
Marriage duration	9.2 ± 4.3	6.6 ± 2.9	0.016	8.1 ± 3.7	6.9 ± 3.4	0.161	8.0 ± 4.3	7.1 ± 3.1	0.289
Infertility duration	5.9 ± 3.2	4.9 ± 2.8	0.179	5.2 ± 3.3	5.2 ± 2.8	0.911	5.4 ± 3.2	5.1 ± 2.9	0.651
Adjustment disorder	40.1 ± 12.5	49.6 ± 13.1	0.005	40.2 ± 14.0	50.0 ± 14.8	0.005	38.2 ± 10.6	49.9 ± 12.4	< 0.001
Hospitalized anxiety and depression	5.3 ± 3.9	14.7 ± 7.5	< 0.001	5.0 ± 3.4	14.9 ± 9.1	< 0.001	5.2 ± 3.6	15.6 ± 8.1	< 0.001
Fertility adjustment scale	40.1 ± 6.7	44.8 ± 7.7	0.016	40.8 ± 6.1	44.1 ± 7.1	0.045	39.9 ± 7.2	44.2 ± 7.4	0.020
Fertility problem inventory	128.7 ± 31.9	150.3 ± 33.4	0.012	126.3 ± 24.7	153.5 ± 38.5	0.001	120.1 ± 30.1	152.4 ± 27.3	< 0.001
Cognitive therapy awareness scale	55.0 ± 16.9	69.3 ± 26.7	0.023	-	-	-	-	-	-
Automatic thought questionnaire	49.6 ± 16.8	63.8 ± 26.6	0.024	-	-	-	-	-	-

The findings indicated that adjustment disorder (*P* = 0.005), total anxiety and depression (*P* < 0.001), fertility adjustment problem (*P* = 0.016), infertility stress (*P* = 0.012), cognitive therapy awareness (*P* = 0.023), and automatic thought (*P* = 0.024) were significantly higher in the nonrespondents in the post-treatment stage. Nevertheless, marriage duration (*P* = 0.016) was higher in the respondents.

Nonrespondents were older and experienced higher levels of adjustment disorder, psychological distress, fertility adjustment problems, and infertility stress compared to the respondents in the 3- and 6-month follow-ups.

Table 2 compares the mean scores of demographic and psychological variables regarding the participants’ response to treatment in the CBT group. The adjustment disorder (*P* < 0.001) and total anxiety and depression (*P* < 0.001) scores were significantly higher in the nonrespondent than the respondent group in the end-of-treatment, 3- and 6-month follow-up periods. Women with higher scores of fertility adjustment problems were less likely to respond to the treatment in the end-of-treatment (*P* = 0.001) and 3-month follow-up (*P* = 0.007) periods.

Infertility stress (*P* < 0.001), cognitive therapy awareness (*P* = 0.003), and automatic thought (*P* = 0.002) scores were higher in the nonresponse group at the end of the intervention. In addition, the nonresponse rate in patients with a high school diploma was lower than those with university education in the 6-month follow-up (*P* = 0.027).

Table 3 outlines predisposing factors associated with nonresponse to treatment in the two intervention groups. Multiple regression logistic analyses were applied to determine the effect of independent variables, including hospital anxiety and depression total score, education level, cause of infertility, principal diagnosis, marriage duration as the predictors of nonresponse to treatment. The results showed that in the ICBT group, longer marriage duration reduced the odds of treatment nonresponse in the post-treatment stage (OR = 0.80, 95% CI: 0.66, 0.97). Participants with the diagnosis of adjustment disorder with mixed anxiety and depressed mood had lower odds of nonresponse than those with the diagnosis of adjustment disorder with depressed mood in the 3-month follow-up (OR = 0.16, 95% CI: 0.03, 0.82). Moreover, the risk of nonresponse was lower in patients with a high school diploma or lower education compared with those with university education in the ICBT group in the 6-month follow-up (OR = 0.05, 95% CI: 0.005, 0.58). Women whose infertility was caused by female factors (OR = 0.03, 95% CI: 0.002, 0.74) and male and female factors (OR = 0.09, 95% CI: 0.01, 0.89) were at reduced odds of nonresponse compared with women whose infertility was due to an unknown factor in ICBT group in the 6-month follow-up. Additionally, patients with a higher level of anxiety and depression symptoms were associated with an increased risk of nonresponse, with the odds ratio of 1.46 (95% CI: 1.18, 1.81) in post-treatment, 1.40 (95% CI: 1.18, 1.67) in 3-month follow-up, and 2.01 (95% CI: 1.35, 2.97) in 6-month follow-up.

**Table 3:** Comparison of the demographic and clinical characteristics of the women facing couple’s fertility problem regarding response to treatment in CBT group

variables	Post-test			3-month follow-up			6-month follow-up		
	Response (n = 24)	non-response (n = 52)	*p*-value	Response (n = 25)	non-response (n = 51)	*p*-value	Response (n = 25)	non-response (n = 51)	*p*-value
Education									
≤Diploma University	11 (45.8) 13 (54.2)	29 (55.8) 23 (44.2)	0.466	15 (60) 10 (40)	25 (49) 26 (51)	0.465	18 (72) 7 (28)	22 (43.1) 29 (56.9)	0.027
Job									
Unemployed Employee	19 (79.2) 5 (20.8)	40 (76.9) 12 (23.1)	1.000	19 (76) 6 (24)	40 (78.4) 11 (21.6)	1.000	21 (84) 4 (16)	38 (74.5) 13 (25.5)	0.398
Place of residence									
City Town	14 (53.8) 10 (41.7)	26 (50) 26 (50)	0.623	13 (52) 12 (48)	27 (52.9) 24 (47.1)	1.000	11 (44) 14 (56)	29 (56.9) 22 (43.1)	0.335
Caused of infertility									
Female factors Male factors Female and male factors Unknown	3 (12.5) 8 (33.3) 5 (20.8) 8 (33.3)	7 (13.5) 15 (28.8) 17 (32.7) 13 (25)	0.722	4 (16) 6 (24) 5 (20) 10 (40)	6 (11.8) 17 (33.3) 17 (33.3) 11 (21.6)	0.283	2 (8) 7 (28) 7 (28) 9 (36)	8 (15.7) 16 (31.4) 15 (29.4) 12 (23.5)	0.621
Principal diagnosis									
depressed mood anxious mood mixed anxiety and depressed mood	11 (45.8) 6 (25) 7 (29.2)	19 (36.5) 15 (28.8) 18 (34.6)	0.742	10 (39.6) 5 (20) 10 (40)	20 (39.2) 16 (31.4) 15 (29.4)	0.507	8 (32) 6 (24) 11 (44)	22 (43.1) 15 (29.4) 14 (27.5)	0.350
Age	34.4 ± 6.4	32.1 ± 5.2	0.103	33.6 ± 6.4	32.5 ± 5.3	0.422	33.5 ± 5.3	32.5 ± 5.9	0.473
Marriage duration	8.6 ± 4.7	8.6 ± 4.8	0.996	9.0 ± 4.5	8.4 ± 4.9	0.641	9.0 ± 5.0	8.4 ± 4.7	0.605
Infertility duration	5.7 ± 3.5	6.5 ± 4.07	0.412	6.4 ± 3.4	6.3 ± 4.1	0.917	6.5 ± 3.5	6.2 ± 4.0	0.801
Adjustment disorder	37.2 ± 9.5	50.1 ± 13.3	< 0.001	39 ± 8.3	51.8 ± 12.4	< 0.001	38.8 ± 5.9	49.8 ± 10.8	< 0.001
Hospitalized anxiety and depression	5.2 ± 3.3	18.1 ± 7.0	< 0.001	7.1 ± 4.3	16.3 ± 7.3	< 0.001	6.7 ± 2.3	16.2 ± 7.7	< 0.001
Fertility adjustment scale	39.2 ± 5.6	45 ± 6.7	0.001	40.5 ± 5.5	44.5 ± 5.9	0.007	42.0 ± 4.6	43.7 ± 7.1	0.205
Fertility problem inventory	128.0 ± 21.5	153.8 ± 28.3	< 0.001	141.1 ± 18.5	150.9 ± 26.3	0.100	141.1 ± 14.2	145.9 ± 30.9	0.364
Cognitive therapy awareness scale	57.1 ± 14.9	72.3 ± 28.6	0.003	-	-	-	-	-	-
Automatic thought questionnaire	51.7 ± 15.7	67.8 ± 27.5	0.002	-	-	-	-	-	-

In the CBT group, longer marriage duration decreased the risk of nonresponse in the post-treatment stage (OR = 0.76, 95% CI: 0.60, 0.97). The risk of nonresponse among patients with a high school diploma or lower education in comparison with those with university education is lower in the 6-month follow-up stages (OR = 0.3, 95% CI: 0.003, 0.40). Besides, there was a decreased odds ratio of nonresponse among participants with higher levels of anxiety and depression at the end of treatment (OR = 0.32, 95% CI: 0.15, 0.71), 3-month (OR = 0.64, 95% CI: 0.52, 0.80), and 6-month (OR = 0.51, 95% CI: 0.35, 0.74) follow-ups.

We also used multiple logistic regression analysis to investigate the predictors associated with the patient’s adherence to treatment. In this model, the 2-group adherence (low and high) was used as the dependent variable, while the hospital anxiety and depression total score, education level, cause of infertility, principal diagnosis, marriage duration were used as independent variables. The results revealed that none of the factors were found to be predictors of low adherence in CBT and ICBT groups (Supplementary Table S2).

## Discussion

The present study aimed to examine predisposing factors of symptomatic nonresponse and low adherence to treatment in a recent trial of therapist-guided ICBT and face-to-face CBT. We found that a high anxiety/depression score before psychotherapy increased the risk of nonresponse to psychotherapies of ICBT/CBT in women facing the couple’s fertility problems from post-treatment to the 6-month follow-up. However, 4 factors, including the diagnosis of adjustment disorder with mixed anxiety and depression, low education level, long marriage duration, and infertility caused by female and male factors, reduced the risk of nonresponse to the two ICBT and CBT psychotherapies.

In the current study, the rate of nonresponders in CBT and ICBT groups was 68.4% and 71.1% at the end of treatment, 67.1% and 61.8% in 3-month follow-up, and 67.1% and 68.4% in 6-month follow-up. The two groups had no significant difference in nonresponse to treatment. Faramarzi et al. (2008), in an RCT performed on 89 infertile women who were experiencing mild to moderate depression, showed that the rate of depression resolution among the three groups was as follows: 50% in the fluoxetine group, 79.3% in the CBT group, and 10% in the control group, which means 50%, 20.7%, and 90%, respectively, did not respond or deteriorated [47]. A previous meta-analysis including 87 studies of CBT for anxiety disorders reported that the overall response rate at post-treatment was 49.5%, which means 50.5% did not respond or deteriorated [48]. Another meta-analysis of an Internet-based intervention for depression revealed that the response rate was 56.19% (43.81% nonresponse or deterioration) [20]. The nonresponse rate also highly depends on the definition of critical clinical change. Many studies apply various diagnostic criteria, while others use the change scores, i.e., the reliable change index [49]. However, due to significant heterogeneity across studies, the response rates differ partly because of how response and nonresponse are defined. In addition, Estimated rates of treatment nonresponse differ across studies due to methodological differences, such as research design, assessment time points, treatment types, and disorders.

Low adherence to treatment was significantly higher in the CBT group than in the ICBT group (29.81% vs. 11.8%). In addition, 76.3% in ICBT and 30.3% in CBT completed all 8 sessions. The treatment adherence rate was higher than the previous study on online interventions for depression, with a 41.5% completion rate found in the whole sample [24]. Still, our findings were comparable to previous research reporting that around 70% of participants completed all therapist-guided ICBT sessions [25, 50]. Clifton et al. (2020) evaluated an Internet-based mind/body program including 10 modules among infertile women. The findings demonstrated that 61% of participants in the treatment group completed module five and 39% module ten [51]. When comparing ICBT and CBT, it was found that ICBT offers greater accessibility to treatment content. Accessing treatment materials at any time and from anywhere can improve adherence. Also, patients may choose to participate in ICBT due to reduced stigma and an increased sense of autonomy.

Our finding demonstrated that the risk of nonresponse was lower in patients with a diagnosis of adjustment disorder with mixed anxiety and depressed mood compared to those with a diagnosis of adjustment disorder with depressed mood. A meta-analysis showed that patients with more severe depression were no more likely to require pharmacotherapy for improvement than those with less severe depression [52]. Nevertheless, our results are not comparable with the previous literature. To the best of our knowledge, this predictor has not been investigated before. Therefore, further studies are needed to determine the impact of this predictor on treatment nonresponse to online intervention.

Of the demographic factors, lower education and longer marriage duration seem to decrease the risk of nonresponse in women facing the couple’s fertility problems. Moreover, infertility caused by female factors and male and female factors reduced the odds of nonresponse compared with an unknown factor. A previous study on women facing couples’ fertility problems revealed that for women who had higher levels of anxiety, higher incomes, an explained infertility diagnosis, and completed 4 or more fertility treatment cycles, online psychoeducational support was more advantageous [53]. In contrast with our finding, research reported that a higher educational level was associated with better treatment outcomes [54]. Still, another study claimed that having a lower educational level was not associated with higher odds of nonresponse [15]. Karyotaki et al. (2018) examined age, sex, educational level, ethnicity, relationship status, employment status, comorbid anxiety, baseline depression severity, previous depressive episodes, medication use, and alcohol use as predictors of response to ICBT for depression. They concluded that participants who were in a relationship, older, and native-born responded better to the treatment [20].

According to our results, no sociodemographic characteristics nor psychological variables influenced the risk of low adherence in our sample. A possible explanation is that this study considered a specific number of predictors of treatment adherence, and other variables that could predict treatment adherence were not included in the study. In line with our findings, Castro et al. (2021) indicated that adherence is not significantly predicted by sociodemographic characteristics and baseline severity of depression [24]. On the contrary, previous studies found that participants’ characteristics, such as educational level, age, or employment, are associated with adherence [28, 29, 55]. Regarding the psychological variables, earlier studies showed that low depression severity at baseline predicted better adherence [56, 57]. However, another study reported that higher adherence was predicted by higher depression and anxiety severity [58].

A key finding of this study is that a high level of anxiety/depression score before psychotherapy increases the risk of nonresponse to psychotherapies of ICBT/CBT by 1.4 to 2 more times in women facing the couple’s fertility problems from post-treatment to 6-month follow-up. Although participants with lower levels of anxiety and depression scores obtained greater reductions in symptoms, this does not mean that those with higher scores did not improve. The present study was the first research to report the predisposing factors of nonresponse and low adherence to Internet-based and face-to-face CBT psychotherapy in the field of infertility. Thus, we have to compare the results with other similar variables or clinical populations. In line with our result, Rozental et al. (2019), in a meta-analysis including 29 RCTs of ICBT, found that participants with higher symptom severity on the primary outcome measure at baseline had increased odds of nonresponse [15]. Hedman et al. (2012) conducted a study on 126 patients with social anxiety disorder and concluded that having lower depressive symptoms predicted better treatment response at 6-month follow-up in Internet- and group-based CBT [59]. Higher symptom severity may indicate more anxiety and depression levels and potential comorbidity, which could result in a poorer response to treatment. In contrast with our findings, previous studies reported that higher baseline depression and anxiety scores predicted better outcomes after ICBT [54, 60, 61]. Another study investigating the outcome predictors of ICBT for panic disorder reported that higher anxiety sensitivity seemed to improve treatment response [62]. In addition, previous studies have found that higher levels of anxiety predict increased depression reduction in CBT [63, 64].

A discussion on the limitations of the study is warranted. First, generalizability may not apply to all primary care clinic settings since patients included in this sample were limited to Babol and only women facing the couple’s fertility problem. Future studies with larger samples and more variability in terms of demographics, sex, geographical regions, clinical characteristics, and therapy processes may be conducted to identify other variables predicting treatment nonresponse and adherence. Second, the way to define nonresponse to treatment and low adherence to the treatment differs across studies, and these criteria are not always strongly correlated. Therefore, it is difficult to interpret the rates of nonresponse and its predictors, especially since there is a lack of consensus on how to define and classify nonresponse patients. Third, our study evaluated a few of the many possible predictors of nonresponse and low adherence to treatment. We analyzed only sociodemographic and psychosocial variables because examining all predictors identified in the literature was beyond the scope of our study. Therefore, evaluating additional factors such as technological usability, intervention design, chronicity of symptoms, and prior treatment experience is needed. Finally, this intervention was conducted in Iran, where access to psychiatric services is limited, and this may have influenced the usage patterns of study participants.

Despite the limitations mentioned above, our study has several strengths. To the best of our knowledge, it was the first study to examine the determinants of nonresponse to treatment and low adherence to therapist-guided ICBT in the field of infertility. The results of this study contributed to the growing evidence about predictors of treatment nonresponse in therapist-guided online interventions among patients with adjustment disorders. Also, primary and secondary outcomes were assessed using validated scales.

Based on prior studies, the prevalence of psychiatric disorders among infertile women is high [65–68]. Ensuring the emotional well-being of patients receiving ARTs is an essential component of comprehensive clinical care. In addition, managing psychological problems such as depression and anxiety may help increase the conception rate [69]. Given that unmanaged infertility anxiety and depression affect responses to fertility treatment, resolving this issue requires more attention and careful planning. Therefore, the proper intervention helps the majority of women facing the couple’s fertility problems to achieve some type of resolution. It has been well-documented that online interventions for infertile individuals can reduce symptoms of anxiety and depression [8, 9, 51, 70]. Besides, identifying factors that influence response to psychological intervention can be highly beneficial. Understanding nonresponders and those with low adherence by psychologists and health providers is crucial for applying the most effective treatments.

Our findings emphasize that higher anxiety and depression symptom severity contributes to impaired outcomes. The current research recommends early recognition and adequate treatment at the symptom onset for gaining a better response. Therefore, these results not only encourage clinicians to use demographic and clinical variables in the early stages of treatment planning but also help track treatment response and patients’ perceptions of treatment. It is also important to keep in mind this does not mean that patients with high anxiety/depression scores, infertility caused by unknown factors, diagnosis of adjustment disorder with depressed mood, higher educational level, and shorter marriage duration cannot achieve improvements. However, patients with high anxiety/depression scores before the treatments should be monitored carefully and receive additional treatment support for remission. This study outlines important psychosocial and demographic factors associated with response to psychotherapy.

### Electronic supplementary material

Below is the link to the electronic supplementary material.

**Supplementary Material 1:** Supplementary Tables S1 and S2

## Data Availability

The data underlying this article will be shared with the corresponding author upon reasonable request.
