# Supplementary material for: Predictors of nonresponse to treatment and low adherence to internet-based cognitive behavioral therapy in depressed/anxious women facing the couple’s fertility problems: a secondary analysis of a randomized control trial
Source: BMC Psychiatry. 2024 Jan 10;24:39. doi: 10.1186/s12888-023-05484-3 (PMC10782627; doi:10.1186/s12888-023-05484-3)
Supplement: Supplementary file 1 — Supplementary Material 1: Supplementary Tables S1 and S2 [file 12888_2023_5484_MOESM1_ESM.docx]

Table S1. The contents of the sessions

| **content** | **session** | **Module** |
| --- | --- | --- |
| Infertility  Causes of infertility  Survival guide for waiting period  Infertility treatments  Stress source in infertility | 1-8 | Psycho-education for infertility |
| Introduction  The emotions and cognition role in emotion disturbances  Automatic thoughts  Connection between thoughts and emotions Identify automatic thought | 1 | Introduction to principles of CBT |
| Changing automatic thoughts  linking thought and emotions | 2 | Restructuring methods |
| Cognitive errors  Cognitive errors in infertility | 3 |  |
| Assessing and developing rational/adaptive thoughts | 4 |  |
| patterns in behavior  Scheduled activities to make the days more pleasant | 5 | Behavioral techniques |
| Exercises to prevent procrastination | 6 |  |
| Determining the schemas (core beliefs) that affect your self-esteem  Determining the schemas associated with infertility  Changing negative core beliefs | 7 | Changing Schemas |
| Relapse prevention  sessions summary  Strategies for addressing problems  Strategies for addressing infertility problems | 8 | Reviewing goals |

Table S2. Multiple regression logistic analyses for non-response to treatment in two groups of psychotherapy

| **variables** | **ICBT** | | | | | | **CBT** | | | | | |
| --- | --- | --- | --- | --- | --- | --- | --- | --- | --- | --- | --- | --- |
|  | **Post-test** | | **3-month follow-up** | | **6-month follow-up** | | **Post-test** | | **3-month follow-up** | | **6-month follow-up** | |
|  | **OR (95% CI)** | **P-value** | **OR (95% CI)** | **P-value** | **OR (95% CI)** | **P-value** | **OR (95% CI)** | **P-value** | **OR (95% CI)** | **P-value** | **OR (95% CI)** | **P-value** |
| Education  ≤Diploma  University | - | - | - | - | 0.05 (0.005, 0.58)  Ref | 0.016 | - | - | - | - | 0.03 (0.003, 0.40)  Ref. | 0.008 |
| Caused of infertility  Female factors  Male factors  Female and male factors  Unknown | - | - | - | - | 0.03 (0.002, 0.74)  4.62 (0.17, 119.65)  0.09 (0.01, 0.89)  Ref | 0.031  0.356  0.039 | - | - | - | - | - | - |
| Principal diagnosis  with depressed mood  with anxious mood  With mixed anxiety and depressed mood | - | - | Ref  0.72 (0.12, 4.11)  0.16 (0.03, 0.82) | 0.717  0.028 | - | - | - | - | - | - | - | - |
| Marriage duration | 0.80 (0.66, 0.97) | 0.030 | - | - |  |  | 0.76 (0.60, 0.97) | 0.028 | - | - | - | - |
| Prior High score of anxiety/depression* | 1.46 (1.18, 1.81) | <0.001 | 1.40 (1.18, 1.67) | <0.001 | 2.01 (1.35, 2.97) | <0.001 | 2.20 (1.41, 3.44) | 0.001 | 1.55 (1.25, 1.91) | <0.001 | 3.01 (1.54, 5.86) | 0.001 |
